# Thread-embedding acupuncture for lumbar herniated intervertebral disc: Protocol for a systematic review and meta-analysis

**DOI:** 10.1097/MD.0000000000017847

**Published:** 2019-11-11

**Authors:** Jin-Young Yoon, Yeon-Cheol Park, Sung-Jin Kim, Bonhyuk Goo, Sang-Soo Nam, Yong-Hyeon Baek, Eun-Jung Kim, Dongwoo Nam, Hyun-Jong Lee, Jae-Soo Kim, Byung-Kwan Seo

**Affiliations:** a Department of Clinical Korean Medicine, Graduate School, Kyung Hee University, 26, Kyungheedae-ro, Dongdaemun-gu, Seoul; b Department of Acupuncture & Moxibustion Medicine, College of Oriental Medicine, Dongguk University, 123, Dongdae-ro, Gyeongju-si, Gyeongsangbuk-do; c Department of Acupuncture & Moxibustion Medicine, College of Korean Medicine, Kyung Hee University, 26, Kyungheedae-ro, Dongdaemun-gu, Seoul; d Department of Acupuncture & Moxibustion medicine, College of Korean medicine, Daegu Haany University, 136, Sincheondong-ro, Suseong-gu, Daegu; e Department of Acupuncture & Moxibustion, Kyung Hee University Hospital at Gangdong, 892, Dongnam-ro, Gangdong-gu, Seoul, Republic of Korea.

**Keywords:** lower back pain, lumbar disc herniation, lumbar herniated intervertebral disc, radiculopathy, thread implantation, thread-embedding acupuncture

## Abstract

**Background:**

Lumbar herniated intervertebral disc (LHIVD) is a frequent disease among patients attending Korean medicine hospitals, and it is associated with considerable medical expenses for the patients. Although several recent randomized clinical trials (RCTs) have reported that thread-embedding acupuncture (TEA) has a more favorable therapeutic effect on LHIVD than other types of acupuncture or other treatments, the evidence remains limited because these trials used poor assessment methods and had a high risk of bias. This study aims to evaluate the evidence for the effectiveness and safety of TEA for LHIVD. In this article, we describe our methods and plan for a systematic review.

**Methods:**

We will conduct an electronic search of the following databases from their inception to May 2018: MEDLINE; EMBASE; COCHRANE; China National Knowledge Infrastructure (CNKI) (a Chinese database); CiNii and J-STAGE (Japanese databases); and KoreaMed, Korean Medical Database (KMbase), Korean Studies Information Service System (KISS), National Digital Science Library (NDSL), Korea Institute of Science and Technology Information (KISTI), Oriental Medicine Advanced Searching Integrated System (OASIS). RCTs investigating any type of TEA will be included. The risk of bias in each study will be evaluated using the Cochrane risk of bias tool. Risk ratios or mean differences with 95% confidence intervals will be used to show the effects of TEA if it will be possible to conduct a meta-analysis. Sensitivity analyses will also be conducted in this study.

**Ethics and dissemination:**

Ethical approval is not necessary as this paper does not involve patient data. The review will be published in a peer-reviewed journal or presented in a conference.

**Trial registration number:**

PROSPERO CRD42019133060.

## Abstract 2

Supplemental Digital Content is available in the text

## 1 Introduction

As the intervertebral disc ages, fibrillation of the fibrous ring can cause circumferential tears and radial fissures. The compression force and the twisting force in this state cause the nucleus pulposus to squeeze out through the fissure, resulting in mechanical pressure on the nerve. This causes lower back pain that radiates to the lower limb; this condition is known as LHIVD.^[1]^ It is caused mainly by minor trauma to the spine, such as that resulting from flexion, extension, rotational movement, and sudden postural changes. However, sometimes the condition can occur without an apparent cause.^[2]^

Depending on the severity of the neurological deficit, either surgical or conservative treatments may be considered.^[3]^ Conservative treatments, such as non-steroidal anti-inflammatory drugs (NSAIDs), epidural injections, physical therapy, and alternative treatments are appropriate for most patients without severe neurological deficits.^[4]^ In addition, there is a growing interest in use of herbal medicine, cupping therapy and various types of acupuncture treatment, focusing on conservative treatment. The proportion of patients using complementary and alternative medicine (CAM) to treat LHIVD is increasing.^[5]^

TEA is special type of acupuncture that inserts medical threads (e.g., catgut or polydioxanone) into subcutaneous tissue or muscles at specific points (e.g., traditional acupuncture points or tender points).^[6]^ Embedding a foreign substance also adds chemical stimulation to the mechanical stimulation of traditional acupuncture.^[7]^ When compared with acupuncture, TEA may produce a strong and long-lasting therapeutic effect.^[8]^ Although several recent RCTs have reported that TEA has a more favorable therapeutic effect on LHIVD than other types of acupuncture or other treatments, the evidence remains limited because these trials used poor assessment methods and had a high risk of bias.^[9]^ In this article, we describe our methods and plan for a systematic review.

## 2 Objectives

This study aims to evaluate the evidence for the effectiveness and safety of TEA for LHIVD.

## 3 Methods

### 3.1 Study registration

The protocol for this review has been registered PROSPERO (CRD42019133060; http://www.crd.york.ac.uk/PROSPERO), and it has been designed according to the Preferred Reporting Items for Systematic reviews and Meta-Analyses Protocols (PRISMA-P) 2015 Statement.

### 3.2 Type of studies

Only RCTs will be included in this review. We will grade these studies as “high” with respect to the risk of bias if detailed descriptions of the randomization process were not provided. Furthermore, if an incorrect randomization method was used, the study will not be included.

### 3.3 Type of participants

Studies with patients who were diagnosed as having LHIVD will be included. The included patients would be aged 18 to 65 years with lower back pain radiating to the lower limbs and with physical examination findings and radiologic (such as MRI or CT) findings consistent with LHIVD.

### 3.4 Types of intervention and comparisons

Studies on the effects of TEA will be included. In studies, the effects of TEA have been compared to those of the other treatment and simple (conventional) treatment.

### 3.5 Data sources

#### 3.5.1 Electronic search

**Figure 1:**
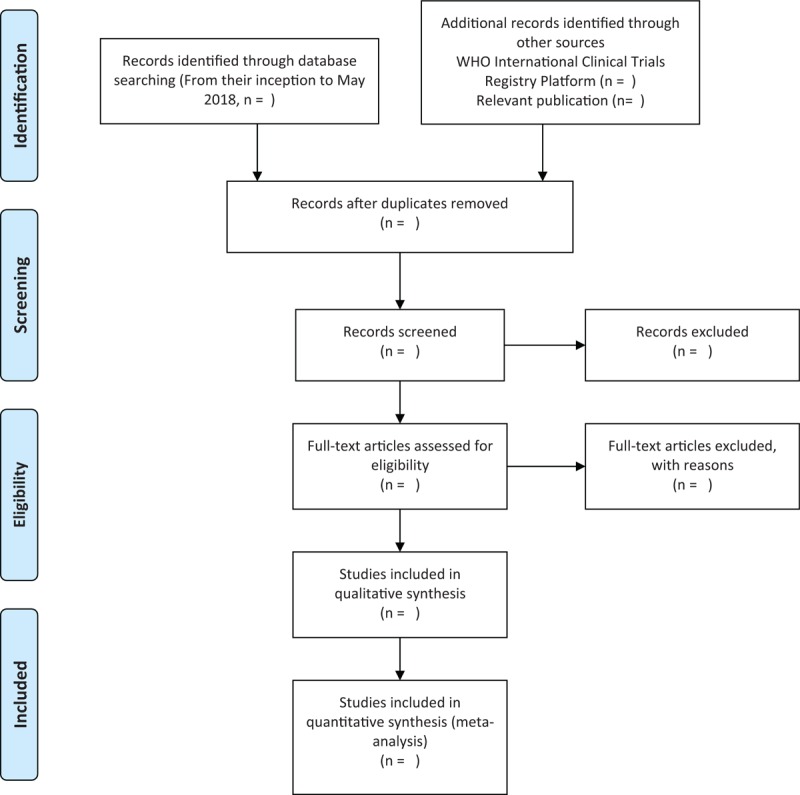
Flow diagram of selection process. The PRISMA flow chart. *From*: Moher D, Liberati A, Tetzlaff J, Altman DG, The PRISMA Group (2009). *Preferred Reporting Items for Systematic Reviews and Meta-Analyses*: The PRISMA Statement. PLoS Med 6(6): e1000097. doi:10.1371/journal.pmed1000097.

The following electronic database will be searched from their inception to May 2018 (Fig. 1): MEDLINE; EMBASE; COCHRANE; China National Knowledge Infrastructure (CNKI) (a Chinese databases); CiNii and J-STAGE (Japanese database); and KoreaMed, Korean Medical Database (KMbase), Korean Studies Information Service System (KISS), National Digital Science Library (NDSL), Korea Institute of Science and Technology Information (KISTI), and Oriental Medicine Advanced Searching Integrated System (OASIS).

The search terms will be a combination of [diagnosis & treatment]. A search on each method of study (meta-analysis, systematic review of literature, randomized clinical study) will be conducted. The search strategy of MEDLINE are shown in Table 1. Additional document shows the detailed search strategies for MEDLINE, CNKI, CiNii, J-STAGE, and the Korean databases. (Appendix 1).

**Table 1:**
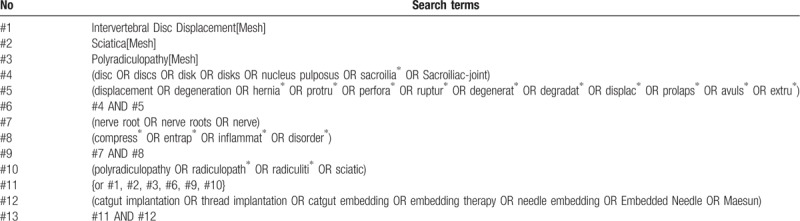
MEDLINE search strategy.

The criteria for the diagnosis of LHIVD was based on a major systematic review.^[10,11]^ First of all, the search terms corresponding to the diagnosis were “HIVD (herniation of intervertebral disc),” “HNP (herniated nucleus pulposus),” “spinal disc herniation,” “lumbar disc herniation,” “intervertebral disc displacement,” “nucleus pulposus hernia,” and “disc degeneration.” For catgut embedding, the search terms were adopted from a related document.^[12]^ Ultimately, we will conduct a search using “catgut embedding,” and “thread implantation.”

#### 3.5.2 Searching other resources

We will search the WHO International Clinical Trials Registry Platform to retrieve recently completed studies. Relevant publications (such as the textbooks on acupuncture and the references within the included studies) will be manually searched. (Fig. 1)

### 3.6 Outcome measures

Pain intensity and functional status/disability will be the outcome measures. The pain will be evaluated using the Visual Analogue Scale (VAS),^[13]^ the numerical rating scale (NRS),^[14]^ or Pain Rating Index (PRI) scale. Functional status/disability will be evaluated using validated measurement tools, such as the Japanese Orthopedic Association (JOA) score and or the Oswestry Disability Index (ODI).^[15]^ Curative rates will be used to evaluate outcomes.

## 4 Analysis

### 4.1 Selection of studies

Two reviewers (SJ and JY) will independently screen the titles and abstracts of the retrieved article lists to exclude any obviously irrelevant articles. The full texts of the remaining articles will be downloaded for assessment for inclusion in the review using predetermined criteria. Disagreements between these two reviewers will be resolved by discussion. If the 2 reviewers will not reach an agreement, a third reviewer (BG) will make the final decision.

### 4.2 Data extraction and management

After conducting the search, two researchers will independently perform the screening procedure. Overlapping studies will be excluded first. Then, studies will be excluded based on the assessment of titles, abstracts, and full texts.

### 4.3 Assessment of the reporting quality and risk of bias

Two reviewers (SJ and JY) will independently assess the risk of bias using the Cochrane Collaboration's “risk of bias” tool. The tool covers six domains: sequence generation, allocation concealment, blinding of participants, blinding of outcome assessors, incomplete outcome data, and selective outcome reporting.^[16]^ The risk of bias for each domain will be rated as “low risk,” “high risk,” or “unclear risk.”

### 4.4 Measures of a treatment effect

The mean difference (MD) or standardized mean difference (SMD) with a 95% confidence interval (CI) will be used to assess the treatment effect for continuous data (such as VAS, NRS, and scores of functional outcome measures). The SMD will be used to estimate a treatment effect when a different outcome scale or method is used. For continuous data, the MD and the 95% CI will be used to estimate a treatment effect when the same outcome scale or method is used. The risk ratio (RR) with a 95% CI will be used to assess the treatment effect for dichotomous data (that is, responder vs non-responder).

### 4.5 Management of missing data

To obtain the missing data, we will contact the corresponding author. If no response will be obtained, we will analyze only the available data and describe the reason and impact of this exclusion in the paper.

### 4.6 Assessment of a reporting bias

Funnel plots will be used to assess publication bias if the number of studies used in the analyses will be sufficient.^[15]^ The Egger regression test will be used to quantitatively evaluate the reporting bias if there will be an asymmetry of the funnel plot.

### 4.7 Assessment of heterogeneity

The heterogeneity between different studies will be quantitatively evaluated using an I^2^ statistic that is derived from *aX*^2^ test. The I^2^ statistic will be calculated to assess the inconsistencies in the results of the included studies. The I^2^ statistic will be interpreted as follows: unimportant heterogeneity, 0% to 40%; moderate heterogeneity, 30%–60%; substantial heterogeneity, 50% to 90%; and considerable heterogeneity, 75% to 100%.^[10]^ If the I^2^ statistic will be >75%, a meta-analysis will not be conducted.^[17]^

### 4.8 Data synthesis and grading of quality of evidence

We will use the Review Manager (REVMAN) software for Windows to perform a meta-analysis and to calculate the RR or SMD (Version 5.3; Copenhagen; The Nordic Cochrane Center, The Cochrane Collaboration, 2014). A random-effects model or a fixed-effect model with a 95% CI will be used to calculate the pooled estimates of the effect size. If we will not be able to conduct a meta-analysis because of lack of clinical studies or because of heterogeneity, we will present the effect size and the 95% CI of every outcome in each clinical trial and describe the significance of important results in the discussion section qualitatively. To summarize the findings of the meta-analysis and describe the strength of evidence, we will use the Grades of Recommendation, Assessment, Development, and Evaluation (GRADE) approach.^[18]^

### 4.9 Sensitivity analysis

After removing the low-quality articles, sensitivity analysis will be conducted to identify the robustness of the results. The methodological quality will be assessed using the “risk of bias” tool.^[19]^ After excluding low-quality articles that have more than three “risk of bias categories” graded as “high risk,” we will conduct a second meta-analysis. The results and effect sizes of the two meta-analyses will be compared and discussed.

## 5 Discussion

LHIVD is the main cause of lower back pain. In 2014, the Ministry of Health and Welfare conducted a survey on the use of CAM treatments. The results showed that LHIVD is the third most common disease among patients using Korean medicine hospitals, accounting for 7.1%. In addition, LHIVD was ranked 10th with respect to the total medical costs in all KM hospitals, accounting for 2.2% of the total medical costs.

The Korea Institute of Oriental Medicine (KIOM) planned an umbrella project on the research progress and development of clinical practice guideline (CPG). As part of this project, the KIOM developed a guideline for LHIVD in 2013; however, this guideline used the Appraisal of Guidelines for Research and Evaluation (AGREE) II.^[20]^ In the most recent amendments to the CPG, published in 2017, some of the existing treatment recommendations were reanalyzed and updated.^[21]^ In 2016, the evidence-based guideline was published, but the review did not include TEA as an intervention.^[22]^ To describe the strength of evidence, this review will use the GRADE approach.^[18]^ The GRADE methodology includes the assessment of the quality of evidence, which includes the risk of bias, inconsistency, immediacy, precision, and effect size. The grade of the recommendation is the degree of certainty that the implementation of an intervention has more benefit than harm. The grade will be determined based on the effect size, level of evidence, and resources.

In this study, we will extend the search period to include the latest studies that have been performed after 2016. We will also increase the number of search terms to obtain the best possible literature through a comprehensive search. This review aims to expand the search strategy and duration, update the references, and introduce usage of the GRADE methodology. In addition, we will expand our search to the Japanese database (CiNii and J-STAGE). The level of evidence in this review will be based on the degree of assurance of the effectiveness of TEA. We anticipate that our review will provide the current clinical evidence on the effectiveness and safety of TEA for LHIVD. We hope it will be useful information to practitioners and patients and be useful for guidance to other countries who are not familiar with the TEA. When designing further clinical research about TEA, this review also may be helpful.

## Author contributions

**Conceptualization:** Byung-Kwan Seo.

**Data curation:** Jae-Soo Kim.

**Methodology:** Jae-Soo Kim.

**Supervision:** Byung-Kwan Seo.

**Writing – original draft:** Jin-Young Yoon, Yeon-Cheol Park.

## Supplementary Material

Supplemental Digital Content

## References

[1] Lee EK Choi EH Lee JE The clinical study on 137 cases of herniated lumbar disc patients. J Korean Acupunct Moxibustion Med Soc 2008;25:127–38.

[2] The Korean Orthopaedic Association. Orthopedics. Seoul: Choisineuihaksa; 1998: 451–4.

[3] Atlas SJ Keller RB Wu YA Long-term outcomes of surgical and nonsurgical management of sciatica secondary to a lumbar disc herniation: 10 year results from the Maine lumbar spine study. Spine 2005;30:927–35. 15834338 10.1097/01.brs.0000158954.68522.2a

[4] Deyo RA Mirza SK Clinical Practice. Herniated lumbar intervertebral disk. N Engl J Med 2016;374:1763–72. 27144851 10.1056/NEJMcp1512658

[5] Ghildayal N Johnson PJ Evans RL Complementary and alternative medicine use in the US adult low back pain population. Glob Adv Health Med 2016;5:69–78. 26937316 10.7453/gahmj.2015.104 PMC4756777

[6] Shin HJ Lee DJ Kwon K The success of thread-embedding therapy in generating hair re-growth in mice points to its possibly having a similar effect in humans. J Pharmacopuncture 2015;18:20–5. 26998386 10.3831/KPI.2015.18.033 PMC4797588

[7] Lee KH Lee DH Kwon KR A literary study on embedding therapy. J Pharmacopunct 2003;6:15–21.

[8] Cho Y Lee S Kim J Thread embedding acupuncture for musculoskeletal pain: a systematic review and meta-analysis protocol. BMJ open 2018;8:e015461. 10.1136/bmjopen-2016-015461 PMC5829818 29374657

[9] Park SH Jeon YT Han KI Literature review of catgut-embedding therapy for lumbar disk herniation. J Korean Med Rehabil 2015;25:29–40.

[10] van der WindtDSimonsERiphagenII Physical examination for lumbar radiculopathy due to disc herniation in patients with low-back pain. Cochrane Database Syst Rev 2010;2:CD007431.10.1002/14651858.CD007431.pub220166095

[11] Rasouli MR Rahimi-Movaghar V Shokraneh F Minimally invasive discectomy versus microdiscectomy/open discectomy for symptomatic lumbar disc herniation. Cochrane Database Syst Rev 2014;9:CD010328. 10.1002/14651858.CD010328.pub2 PMC10961733 25184502

[12] Yun YH Choi IH An opinion about english inscription of thread-embedding therapy. J Korean Med Ophthalmol Otolaryngol Dermatol 2015;28:169–71.

[13] Crichton N Information point: visual analogue scale (VAS). 2001;10:697–706.

[14] Farrar JT Young JP LaMoreaux L Clinical importance ofchanges in chronic pain intensity measured on an 11-point numerical pain rating scale. Pain 2001;94:149–58. 11690728 10.1016/S0304-3959(01)00349-9

[15] Roland M Morris R A study of the natural history of back pain.part I: development of a reliable and sensitive measure of disability in lowback pain. Spine 1983;8:141–4. 6222486 10.1097/00007632-198303000-00004

[16] Higgins JPT Green S Cochrane Handbook for Systematic Reviews of Interventions Version 5.1.0: updated March 2011. The Cochrane Collaboration; 2011.

[17] Lee S Park J Kim J Acupuncture for postoperative pain in laparoscopic surgery: a systematic review protocol. BMJ Open 2014;4:e006750. 10.1136/bmjopen-2014-006750 PMC4275696 25537788

[18] John Wiley & Sons, Higgins JPT Green S Cochrane Handbook for Systematic Reviews of Interventions. 2011.

[19] Higgins JP Altman DG Gøtzsche PC The Cochrane Collaboration's tool for assessing risk of bias in randomised trials. BMJ 2011;343:d5928. 22008217 10.1136/bmj.d5928 PMC3196245

[20] Choi TY Choi J Lee JA The quality of clinical practice guidelines in traditional medicine in Korea: appraisal using the AGREE II instrument. Implement Sci 2015;10:104. 26216349 10.1186/s13012-015-0294-1 PMC4515911

[21] Korea Institute of Orienstal Medicine. The Society of Korean Medicine a Rehabilitation. Korean medicien clinical practice guideline-Lumbar herniated Intervertebral Disc. Korea: Seoul; 2017.

[22] Jun JH Ji Hee Korean medicine clinical practice guideline for lumbar herniated intervertebral disc in adults: An evidence based approach. Eur J Integr Med 2017;9:18–26.

