# Supplementary material for: Thread-embedding acupuncture for lumbar herniated intervertebral disc: Protocol for a systematic review and meta-analysis
Source: Medicine (Baltimore). 2019 Nov 11;98(45):e17847. doi: 10.1097/MD.0000000000017847 (PMC6855608; doi:10.1097/MD.0000000000017847)
Supplement: Supplemental Digital Content [file medi-98-e17847-s001.docx]

**1. Foreign database**

**1.1. Search strategy**

The search terms will be a combination of [diagnosis (OR analogue) AND treatment (OR analogue)] through advanced search (search manager and others). The cross-language search method was used in the CNKI database and was limited to core journals.

**1.2. Search term for each intervention**

**1.2.1. Thread embedding therapy**

MEDLINE - PubMed

#1 Intervertebral Disc Displacement[Mesh]

#2 Sciatica[Mesh]

#3 Polyradiculopathy[Mesh]

#4 (disc OR discs OR disk OR disks OR nucleus pulposus OR sacroilia*

OR Sacroiliac-joint)

#5 (displacement OR degeneration OR hernia* OR protru* OR perfora* OR ruptur* OR degenerat* OR degradat* OR displac* OR prolaps* OR avuls* OR extru*)

#6 #4 AND #5

#7 (nerve root OR nerve roots OR nerve)

#8 (compress* OR entrap* OR inflammat* OR disorder*)

#9 #7 AND #8

#10 (polyradiculopathy OR radiculopath* OR radiculiti* OR sciatic)

#11 {or #1, #2, #3, #6, #9, #10}

#12 (catgut implantation OR thread implantation OR catgut embedding OR embedding therapy OR needle embedding OR Embedded Needle OR Maesun)

#13 #11 AND #12

CENTRAL - Cochrane

#1 Intervertebral disk displacement[ti,ab,kw]

#2 sciatica[ti,ab,kw]

#3 polyradiculopathy[ti,ab,kw]

#4 (disc OR discs OR disk OR disks OR nucleus pulposus OR sacroilia* OR Sacroiliac-joint)[ti,ab,kw]

#5 (displacement OR degeneration OR hernia* OR protru* OR perfora* OR ruptur* OR degenerat* OR degradat* OR displac* OR prolaps* OR avuls* OR extru* )[ti,ab,kw]

#6 #4 AND #5

#7 (nerve root OR nerve roots OR nerve)[ti,ab,kw]

#8 (compress* OR entrap* OR inflammat* OR disorder*)[ti,ab,kw]

#9 #7 AND #8

#10 (polyradiculopathy OR radiculopath* OR radiculiti* OR sciatic)[ti,ab,kw]

#11 {or #1-#3, #6, #9, #10}

#12 (catgut implantation OR thread implantation OR catgut embedding OR embedding therapy OR needle embedding OR Embedded Needle OR Maesun)[ti,ab,kw]

#13 #11 AND #12

EMBASE

#1 ‘Intervertebral disk displacement’/exp

#2 ‘sciatica’/exp

#3 ‘polyradiculopathy’/exp

#4 (disc OR discs OR disk OR disks OR nucleus pulposus OR sacroilia* OR Sacroiliac-joint):ab,ti

#5 (displacement OR degeneration OR hernia* OR protru* OR perfora* OR ruptur* OR degenerat* OR degradat* OR displac* OR prolaps* OR avuls* OR extru* ):ab,ti

#6 #4 AND #5

#7 (nerve root OR nerve roots OR nerve):ab,ti

#8 (compress* OR entrap* OR inflammat* OR disorder*):ab,ti

#9 #7 AND #8

#10 (polyradiculopathy OR radiculopath* OR radiculiti* OR sciatic):ab,ti

#11 {or #1-#3, #6, #9, #10}

#12 (catgut implantation OR thread implantation OR catgut embedding OR embedding therapy OR needle embedding OR Embedded Needle OR Maesun):ab,ti

#13 #11 AND #12

CNKI

“HIVD” OR “Herniated Intervertebral disc” OR “HNP” OR “Herniated nucleus pulposus” OR “spinal disc herniation” OR “intervertebral disc herniation” OR “herniated disc” OR “Intervertebral disc displacement”

AND

"catgut implantation" OR "thread implantation" OR "catgut embedding" OR "embedding therapy" OR "needle embedding" OR "Embedded Needle" OR "Maesun“

CiNII and J-Stage

((((((((椎間板 OR 神経根症 OR ディスク) OR (Intervertebral Disc Displacement OR Sciatica) OR Polyradiculopathy) OR ((((disc OR discs OR disk OR disks OR nucleus pulposus OR sacroilia* OR Sacroiliac-joint))) AND ((displacement OR degeneration OR hernia* OR protru* OR perfora* OR ruptur* OR degenerat* OR degradat* OR displac* OR prolaps* OR avuls* OR extru*)))) OR ((((nerve root OR nerve roots OR nerve))) AND ((compress* OR entrap* OR inﬂammat* OR disorder*)))) OR (((polyradiculopathy OR radiculopath* OR radiculiti* OR sciatic))))

AND (catgut implantation" OR "thread implantation OR catgut embedding OR embedding therapy OR needle embedding OR Embedded Needle OR Maesun OR 埋線)

**2. Domestic database**

**2.1. Search strategy**

The search terms will be a combination of [diagnosis(OR analogue) AND treatment(OR analogue)]. Searches will be performed by combining search terms through detailed searches supported by each database.

**2.2. Search terms for each intervention**

**2.2.1. Thread embedding therapy**

KoreaMed

((disc[ALL] OR nucleus pulposus[ALL]) AND (displacement[ALL] OR degeneration[ALL] OR hernia[ALL] ))

AND

(매선[ALL] OR catgut[ALL] OR thread implantation[ALL] OR embedding therapy[ALL] OR needle embedding[ALL])

한국의학논문데이터베이스 (KMbase)

((([ALL=disc] OR [ALL=nucleus pulposus]) AND ([AHLL=displacement] OR [ALL=degeneration] OR [ALL=hernia*])) OR [ALL=추간판탈출증] OR [ALL=디스크탈출증]) AND ([ALL=매선] OR [ALL=catgut] OR [ALL=thread implantation] OR [ALL=embedding therapy] OR [ALL=needle embedding])

한국학술정보 (KISS)

(((disc OR nucleus pulposus) AND (displacement OR degeneration OR hernia*)) OR (추간판탈출증 OR 디스크탈출증) AND (매선 OR catgut OR thread implantation OR embedding therapy OR needle embedding)

과학기술정보통합서비스 (NDSL)

(((disc OR nucleus pulposus) AND (displacement OR degeneration OR hernia*)) OR (추간판탈출증 OR 디스크탈출증) AND (매선 OR catgut OR thread implantation OR embedding therapy OR needle embedding)

과학기술학회마을 (KISTI)

(BI: disc hernia*) OR (BI: nucleus pulposus hernia*) OR (BI: disc displacement) OR (BI: nucleus pulposus displacement) OR (BI: disc degeneration) OR (BI: nucleus pulposus degeneration) OR (BI: 추간판탈출증) OR (BI: 디스크탈출증) AND (BI: 매선) OR (BI: catgut) OR (BI: thread implantation) OR (BI: embedding therapy) OR (BI: needle embedding)

OASIS

(((disc OR nucleus pulposus) AND (displacement OR degeneration OR hernia*)) OR (추간판탈출증 OR 디스크탈출증) AND (매선 OR catgut OR thread implantation OR embedding therapy OR needle embedding)
